# Supplementary figures and images for: Maize adaptation across temperate climates was obtained via expression of two florigen genes
Source: PLoS Genet. 2020 Jul 16;16(7):e1008882. doi: 10.1371/journal.pgen.1008882 (PMC7386623; doi:10.1371/journal.pgen.1008882)

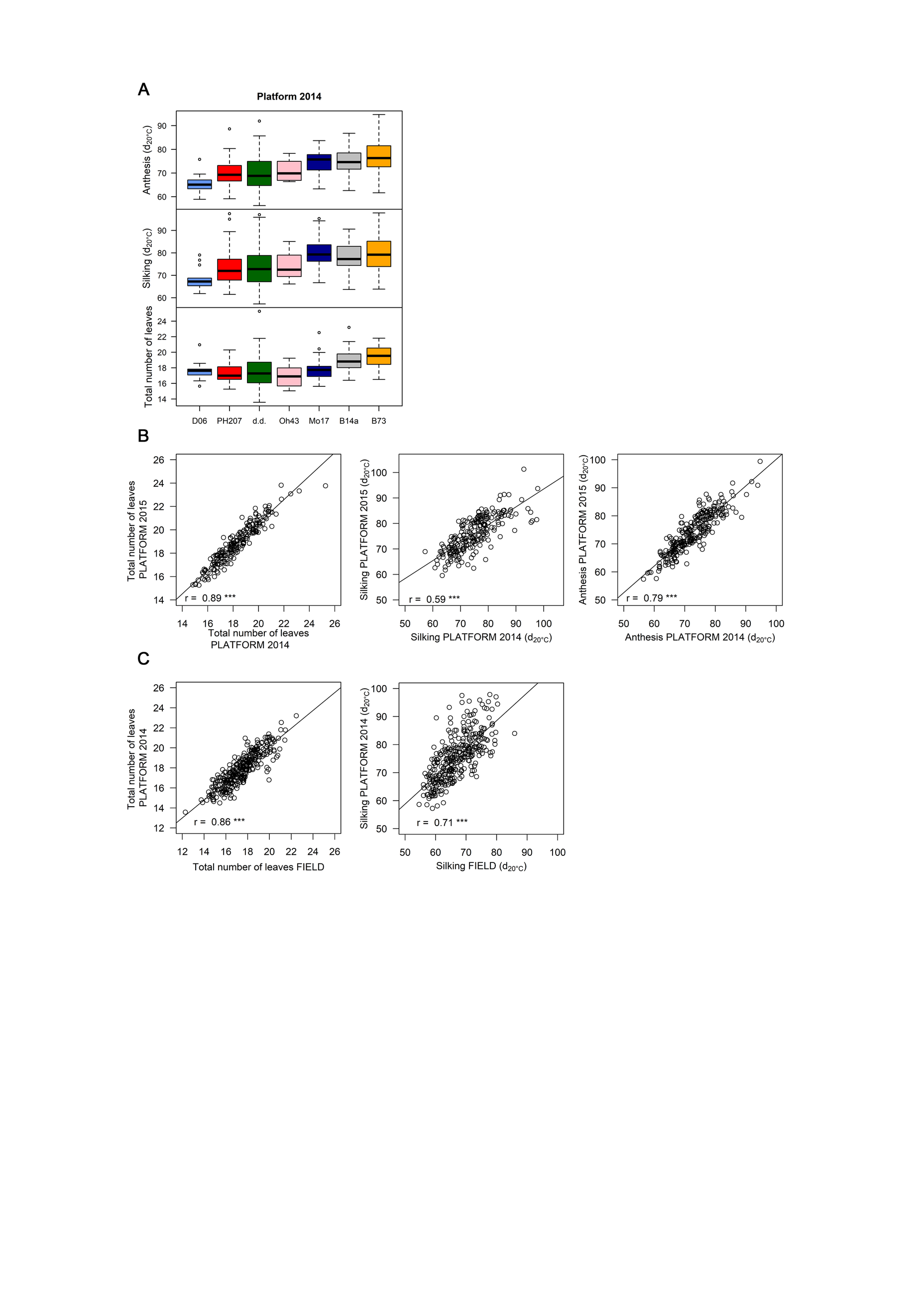

Supplement: S1 Fig — (A) Genotypic values of anthesis time, silking and total number of leaves per genetic group, during the platform experiment in 2014. Genetic groups are represented by the name of their founder. D06: n = 14; PH207: n = 42; diverse dents (d.d.): n = 128; Oh43: n = 16; Mo17: n = 35; B14a: n = 41; B73: n = 37. (B) Relationship between genotypic values of the flowering traits (respectively anthesis, silking and total number of leaves) in platform experiments conducted over two consecutive years (2014 and 2015). n = 273 lines in common between both years. (C) Relationship between genotypic values for silking (left panel) and the total number of leaves (right panel) measured in the platform (2014) and field (2015–2016). n = 313 lines. In (A), (B) and (C), genotypic values are BLUEs. Correlation coefficients are displayed together with their significance (*** p < 10−4). (TIF) [file pgen.1008882.s001.tif]

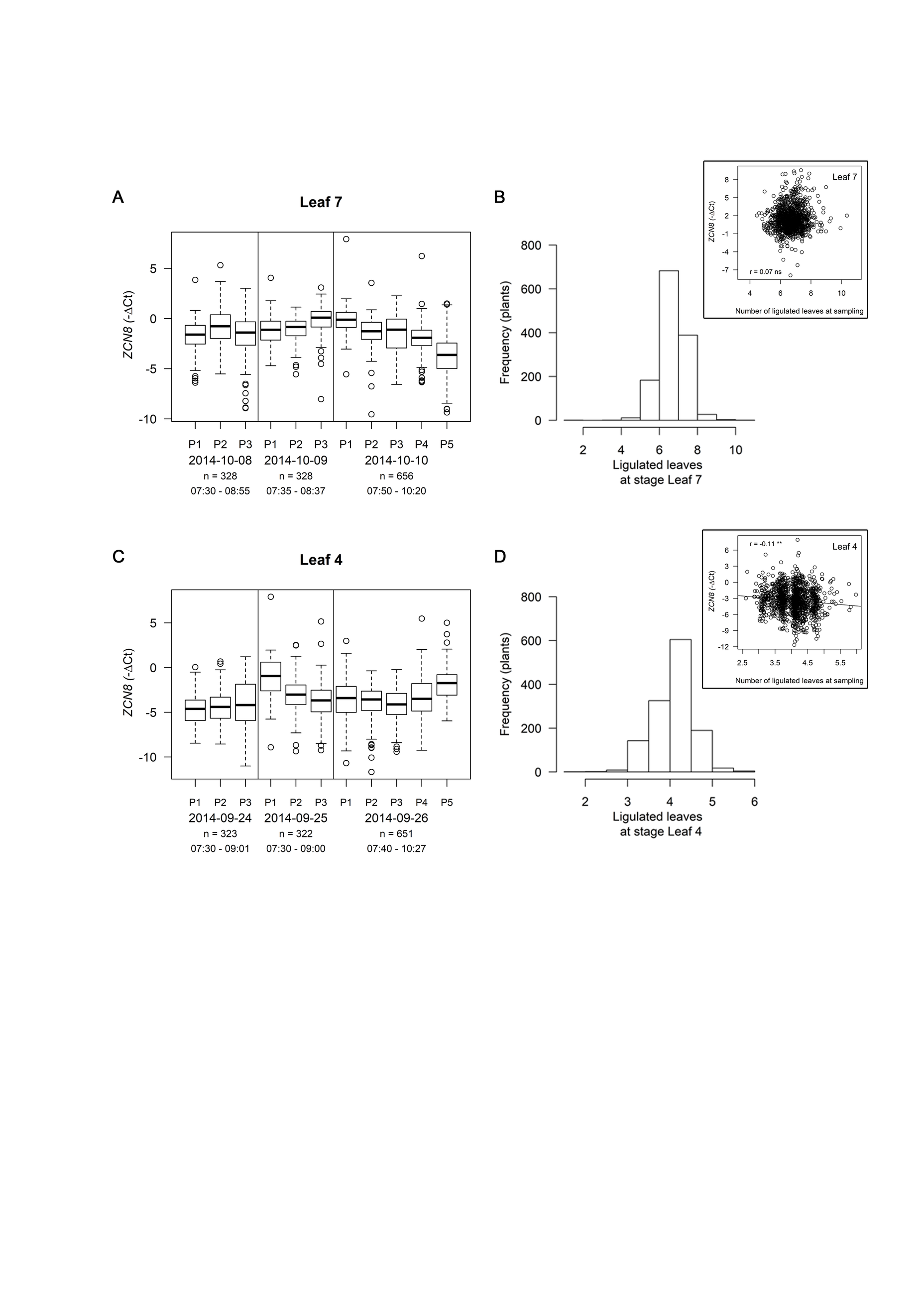

Supplement: S2 Fig — (A, C) ZCN8 transcript accumulation over three consecutive days per sub-period of ca. 30 minutes (covering 1.5 to 3 hours after dawn within each sampling day) at stage ligulated leaf 7 (A) or 4 (C). The number of individuals that were sampled is indicated for each day together with the time window of sampling. (B, D) Distribution of the actual number of ligulated leaves within the population at the sampling stage ligulated leaf 7 (B) or 4, (D). Insets show the absence of correlation between ZCN8 transcript accumulation and the actual number of ligulated leaves. (TIF) [file pgen.1008882.s002.tif]

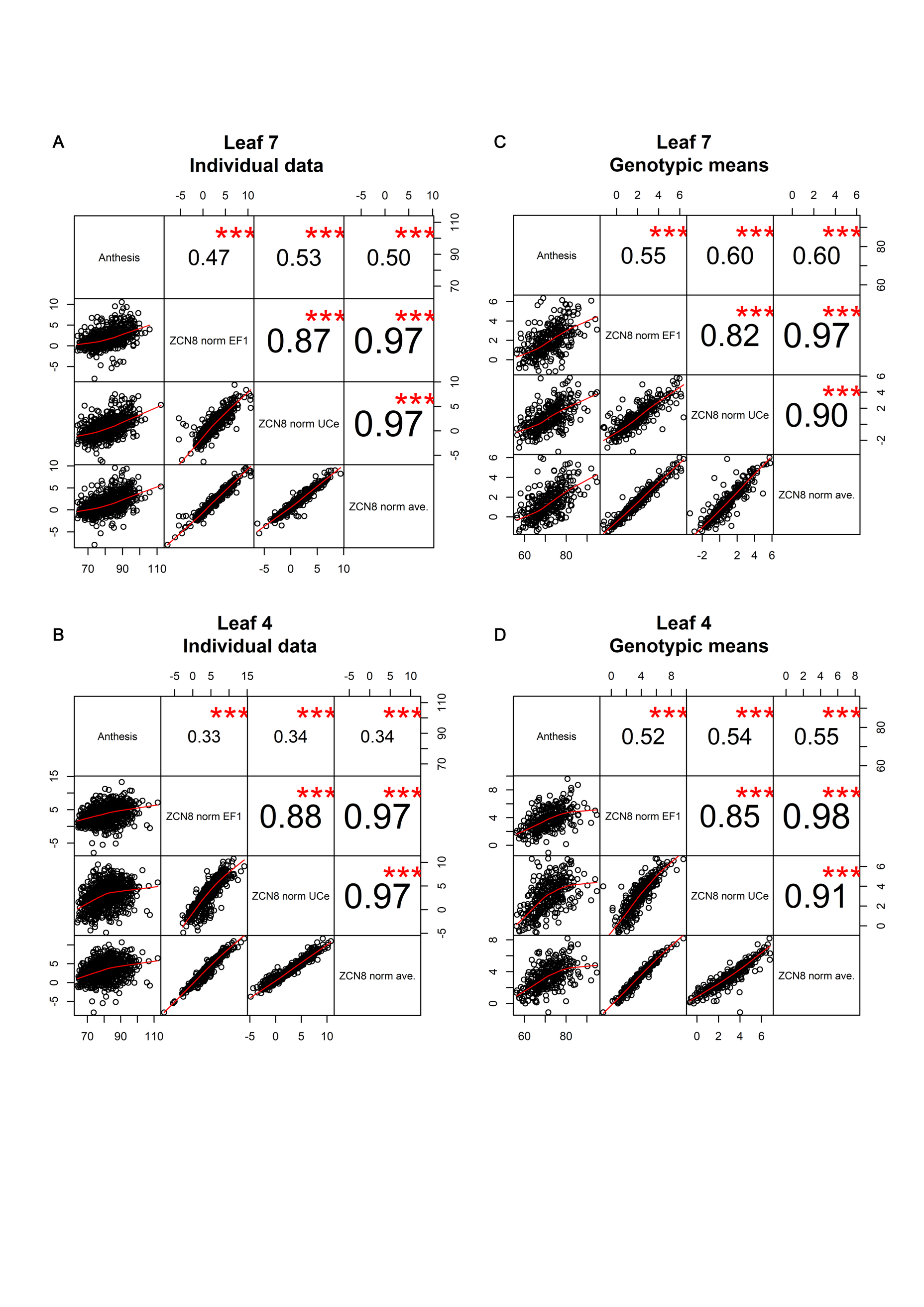

Supplement: S3 Fig — (A, B) Correlation between anthesis and expression of ZCN8 transcript accumulation—respectively measured at stage leaf 7 (A) or leaf 4 (B)—normalized either by EF1a, by UCe, or by the average (“ave”) of EF1a and UCe transcript accumulations, for individual data (n = 980 plants). (C, D) Correlation between anthesis and expression of ZCN8 transcript accumulation—respectively measured at stage leaf 7 (C) or leaf 4 (D)—normalized against EF1a, UCe, or the average (“ave”) of EF1a and UCe transcripts accumulation, for genotypic values (n = 320 lines). In each panel, the X and Y axes show the units of the variable displayed in the main diagonal. Transcript accumulation is expressed as ΔCt. Anthesis time expressed in equivalent days at 20°C after germination Pearson’s correlation coefficients are indicated with their significance (***, p < 10−4). (TIF) [file pgen.1008882.s003.tif]

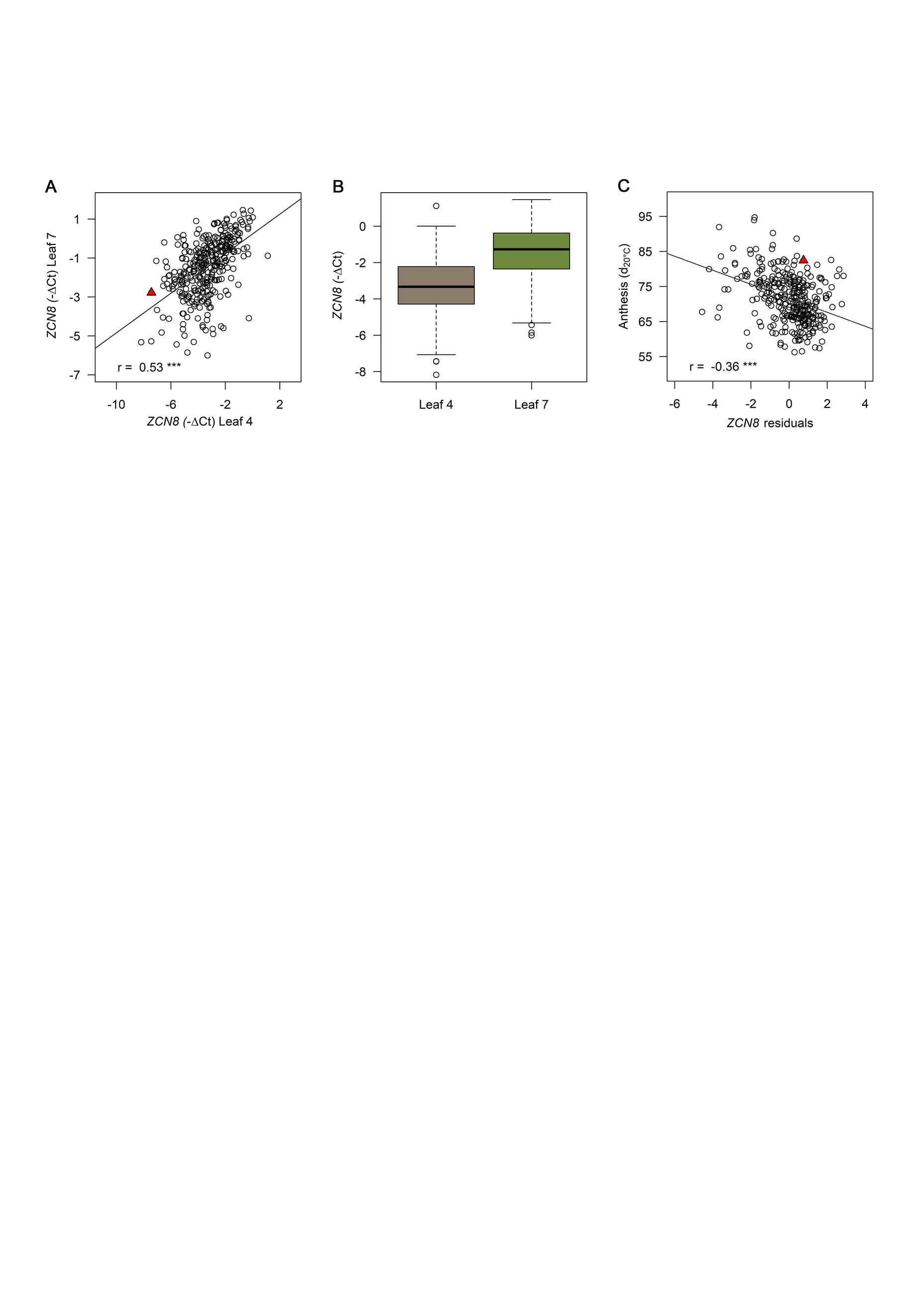

Supplement: S4 Fig — (A, B) Genotypic values of ZCN8 accumulation at stages ligulated leaf 4 and 7 in the platform experiment of 2014. n = 320 lines. The effect of stage (L4 vs L7) on ZCN8 accumulation was found significant (p < 0.001) by ANOVA. (C) Relationship between the residuals extracted from the regression between ZCN8 at stage ligulated leaf 4 vs. 7 displayed in (A), and anthesis time measured in the same platform experiment. Genotypic values are BLUEs. Pearson’s correlation coefficients are indicated with their significance (***, p < 10−4). (TIF) [file pgen.1008882.s004.tif]

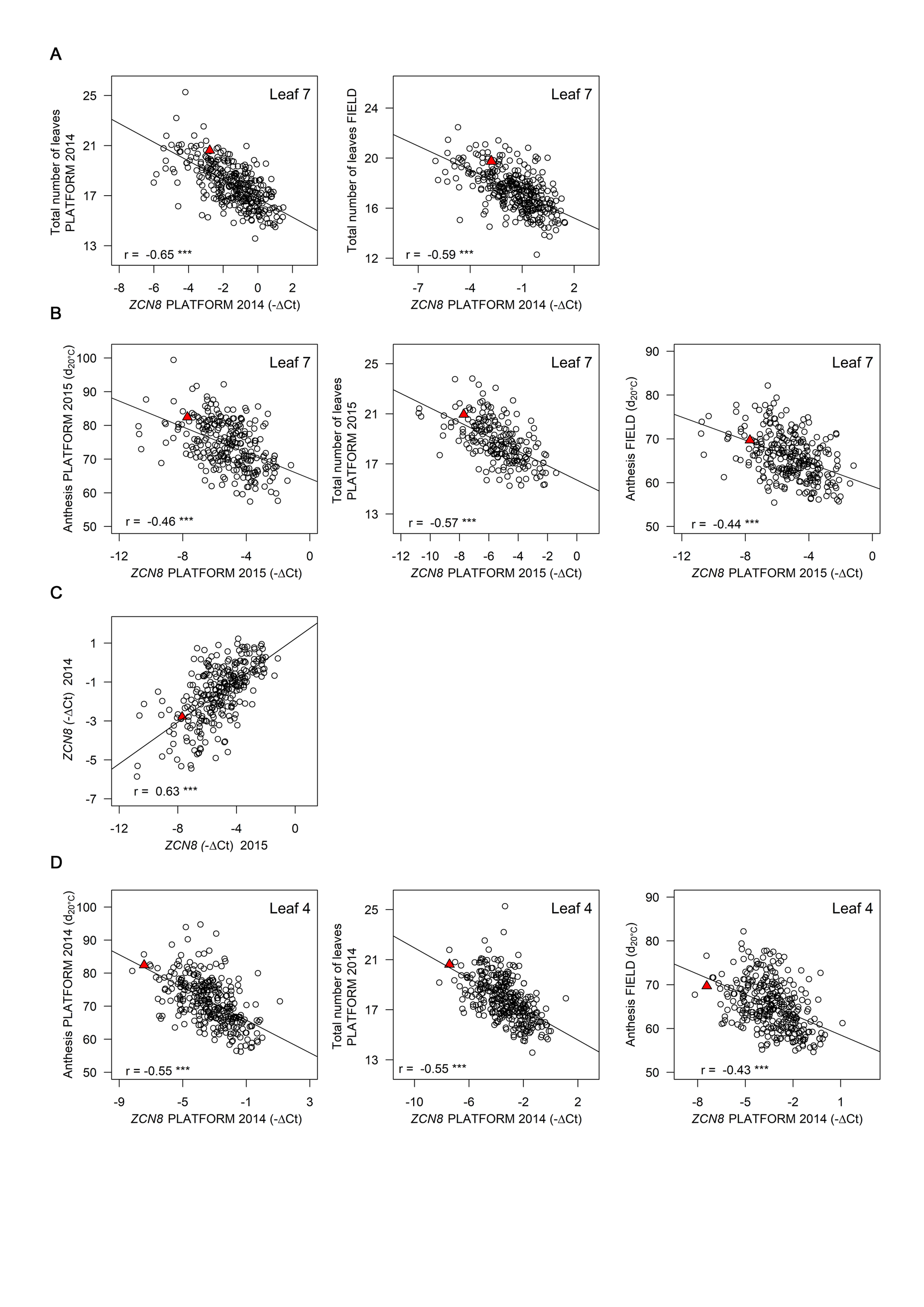

Supplement: S5 Fig — (A) Relationship between genotypic values of ZCN8 accumulation measured at stage ligulated leaf 7 in the platform in 2014 and the total number of leaves respectively measured in the same experiment (left panel) or the field in 2015–2016 (right panel). n = 313 lines. (B) Relationship between genotypic values of ZCN8 accumulation in the platform experiment of 2015 on a subset of lines (n = 273 lines) and flowering traits respectively measured during the same experiment (left panel, anthesis; middle panel, total number of leaves) or in the field in 2015–2016 (anthesis, right panel). (C) Relationship between genotypic values of ZCN8 accumulation in platform experiments of 2014 and 2015. n = 273 lines. (D) Relationship between genotypic values of ZCN8 accumulation measured at stage ligulated leaf 4 in the platform (2014) and flowering traits either measured in the same experiment (left panel, anthesis; middle panel, total number of leaves) or the field (right panel, anthesis). n = 313 lines. Genotypic values are BLUEs. Pearson’s correlation coefficients are indicated with their significance (***, p < 10−4). (TIF) [file pgen.1008882.s005.tif]

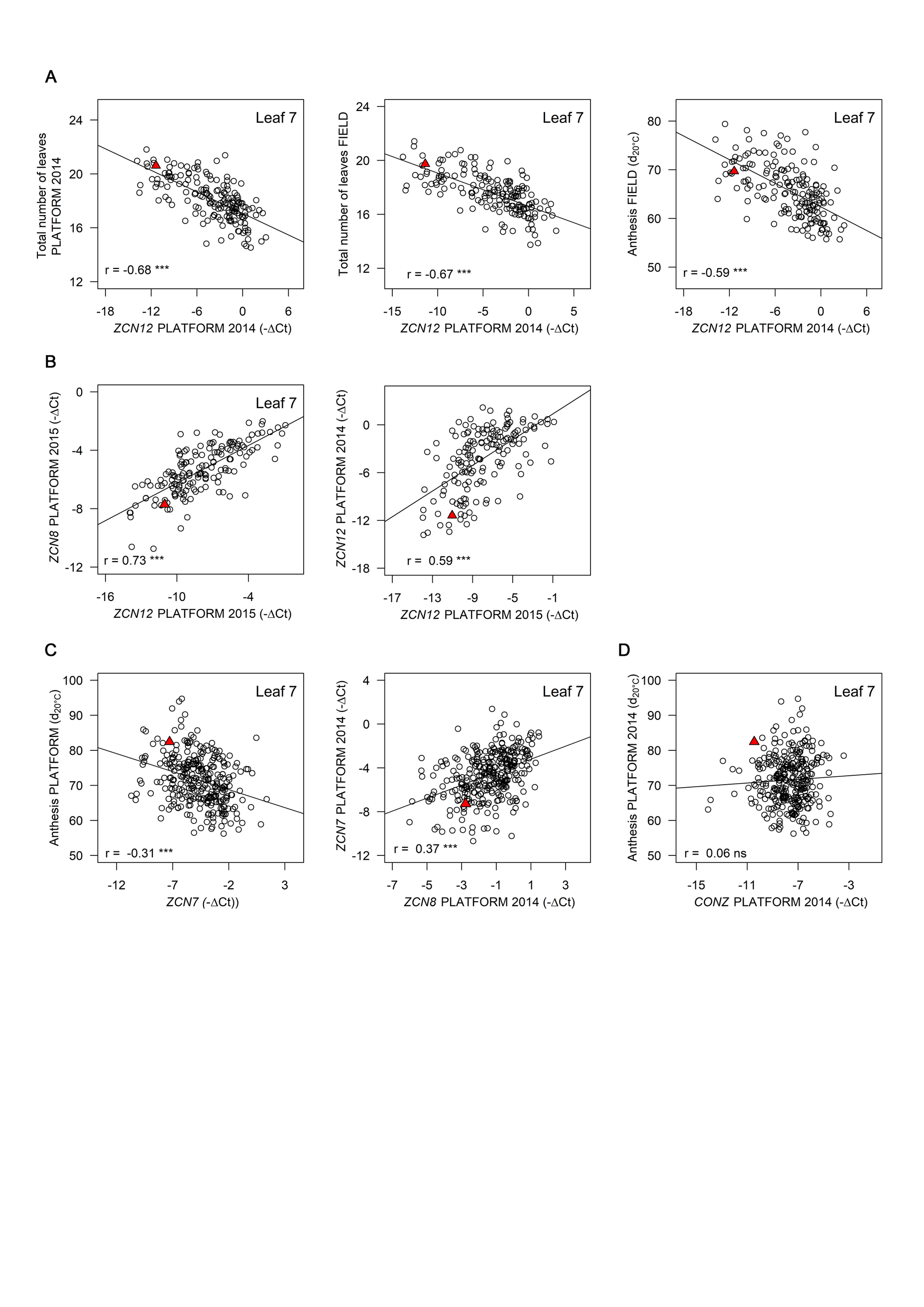

Supplement: S6 Fig — (A) Relationship between genotypic values of ZCN12 accumulation measured at stage ligulated leaf 7 in the platform in 2014 and flowering traits measured in the same experiment (total number of leaves, left panel) or the field in 2015–2016 (total number of leaves and anthesis, middle and right panels). n = 173 lines. (B) Relationship between genotypic values of ZCN12 accumulation in the platform experiment of 2015 on a subset of lines (n = 160 lines) and, respectively, accumulation of ZCN8 measured during the same experiment (left panel) or accumulation of ZCN12 measured in the first platform experiment (right panel). (C) Relationship between genotypic values of ZCN7 and anthesis time (left panel) and ZCN8 accumulation (right panel) in the platform in 2014. (D) Relationship between genotypic values of anthesis measured in the platform in 2014 and ZmCONZ1 accumulation. Genotypic values are BLUEs. Pearson’s correlation coefficients are indicated with their significance (***, p < 10−4). The red triangle represents genotypic values for the reference line B73. (TIF) [file pgen.1008882.s006.tif]

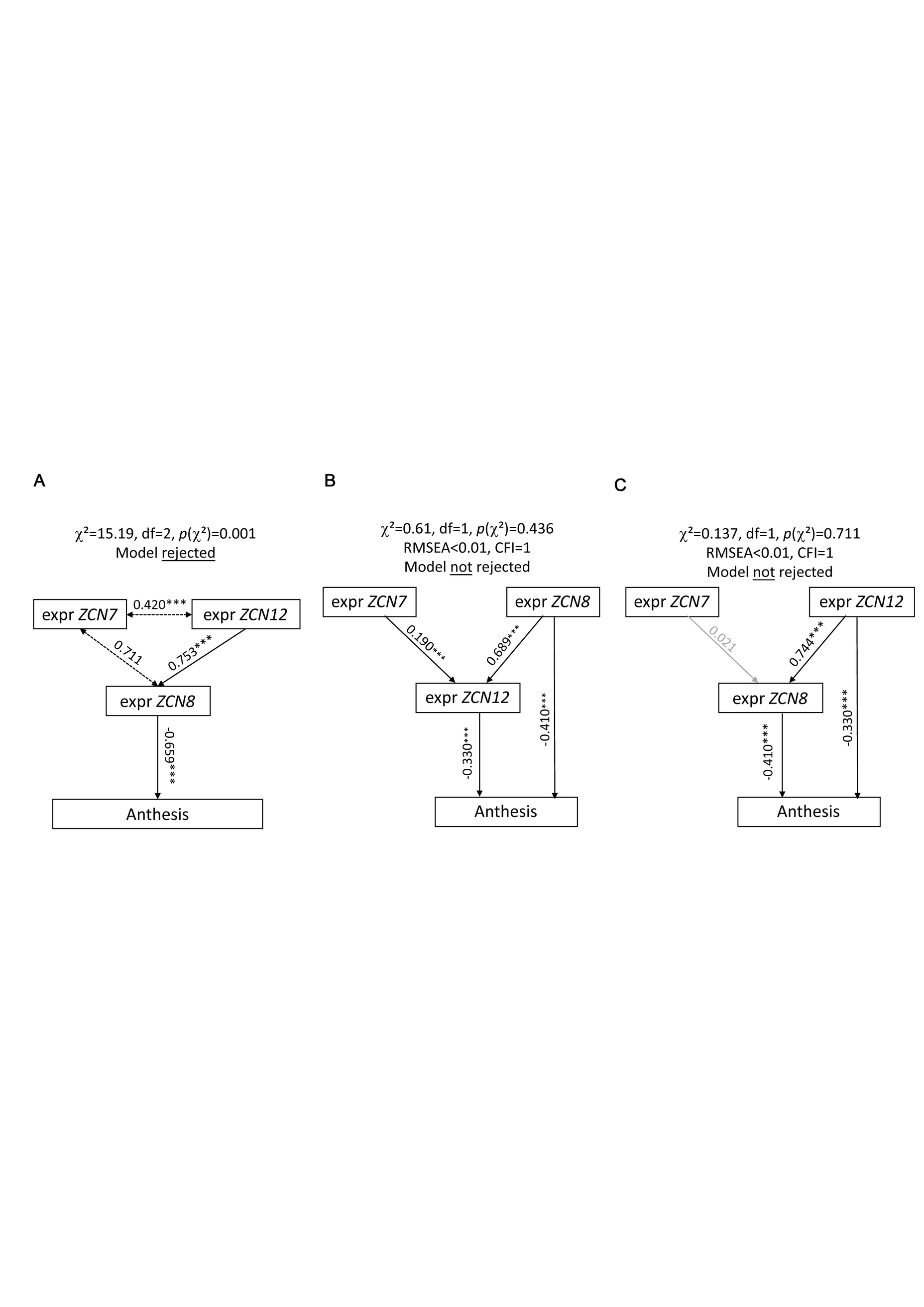

Supplement: S7 Fig — Models in which ZCN12 has an indirect effect on anthesis (A) were rejected, whereas models in which both ZCN8 and ZCN12 directly affect anthesis were accepted, with an indirect effect of ZCN7 (B and C). Arrows represent linear functional relationships between anthesis and florigen expression. Simple-headed arrows represent causal relationships, and double-headed arrows represent free correlations. Standardized path coefficients are indicated on each arrow with their level of significance (***: P < 0.001, ns: not significant). All three models were tested against our data on BLUEs and results are given in the upper part of each panel. See also Fig 3. (TIF) [file pgen.1008882.s007.tif]

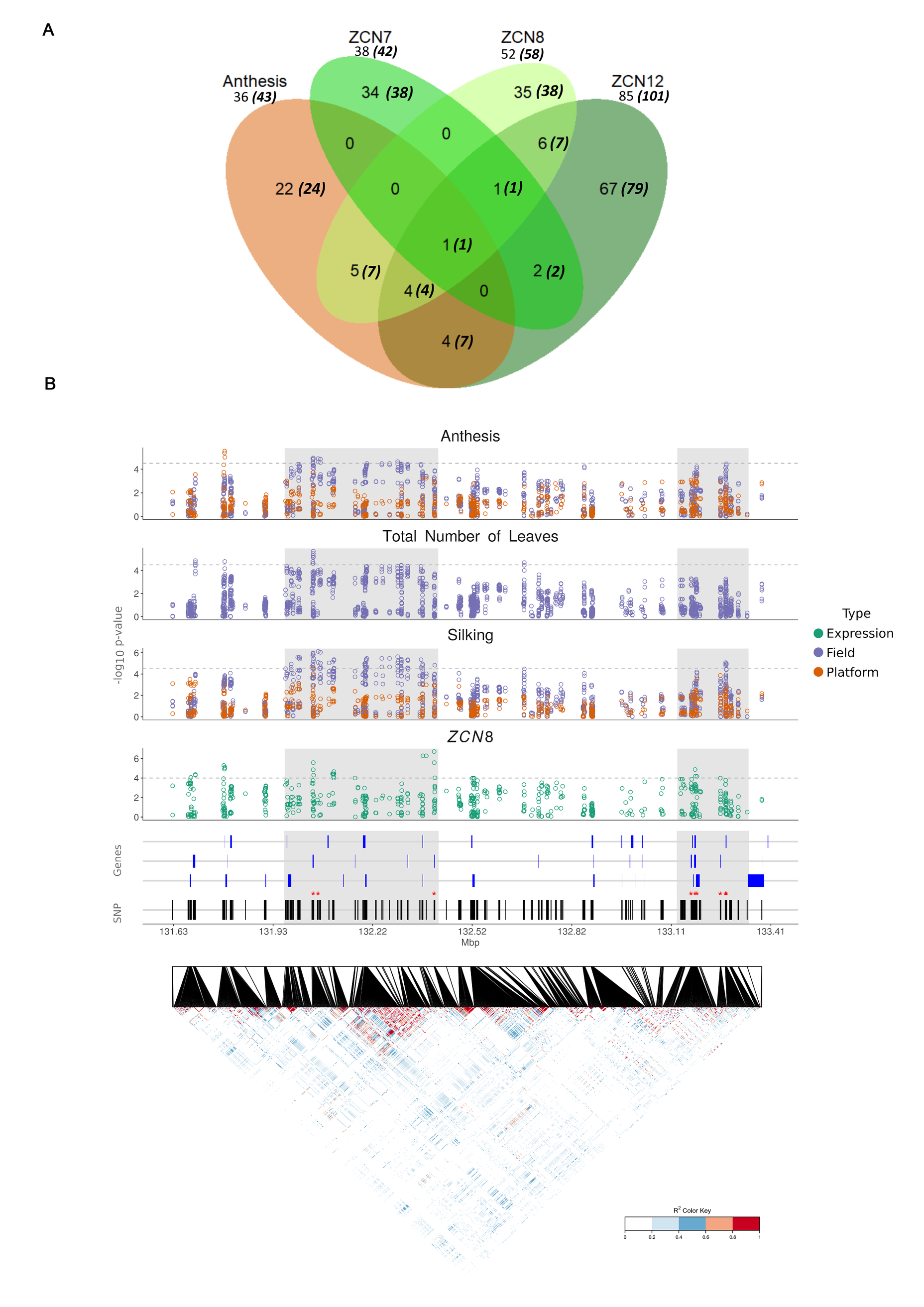

Supplement: S8 Fig — (A) Number of QTLs shared between anthesis in the platform experiment (2014) and ZCN eQTLs. Anthesis was recorded in the platform in 2014 and ZCN8/7/12 levels were measured in the platform, in 2014 and 2015 at different leaf stages. GWAS was conducted for every trait/experiment, and significant SNPs were grouped according to genetic distances, with a threshold at 0.1 cM to define QTLs. The number of these QTLs is italicised within brackets outside of VENN diagram. The number of common QTLs between traits is italicised within brackets inside each VENN area. QTLs detected were then gathered into ‘meta-QTLs’ regions containing overlapping individual QTLs. The number of common ‘meta-QTLs’ is shown in plain text outside and inside the circles, and a common ‘meta-QTL’ is defined as regions containing at least one QTL of each trait considered. (B) Regional association plot for one meta QTL on chromosome 8. Distribution of the -log10(pval) for all variants in the region. The dotted grey line corresponds to–log10(pval) = 4.5 for anthesis-related traits evaluated in the field (purple) and platform (orange) and 4 for the expression traits evaluated in the platform (green). Bottom panel, linkage disequilibrium (LD) heat map of all SNPs in the QTL showing the local LD (r2) between all the variants; Black lines represent the distribution of the SNPs and the blue boxes represents the genes mapped for the region. Red asterisks represent the position of the most significant SNPs. (See also S4 Table for candidate genes). Grey areas represent sub-regions harbouring the most significant SNPs and genes and having low LD between them on average. (TIF) [file pgen.1008882.s008.tif]

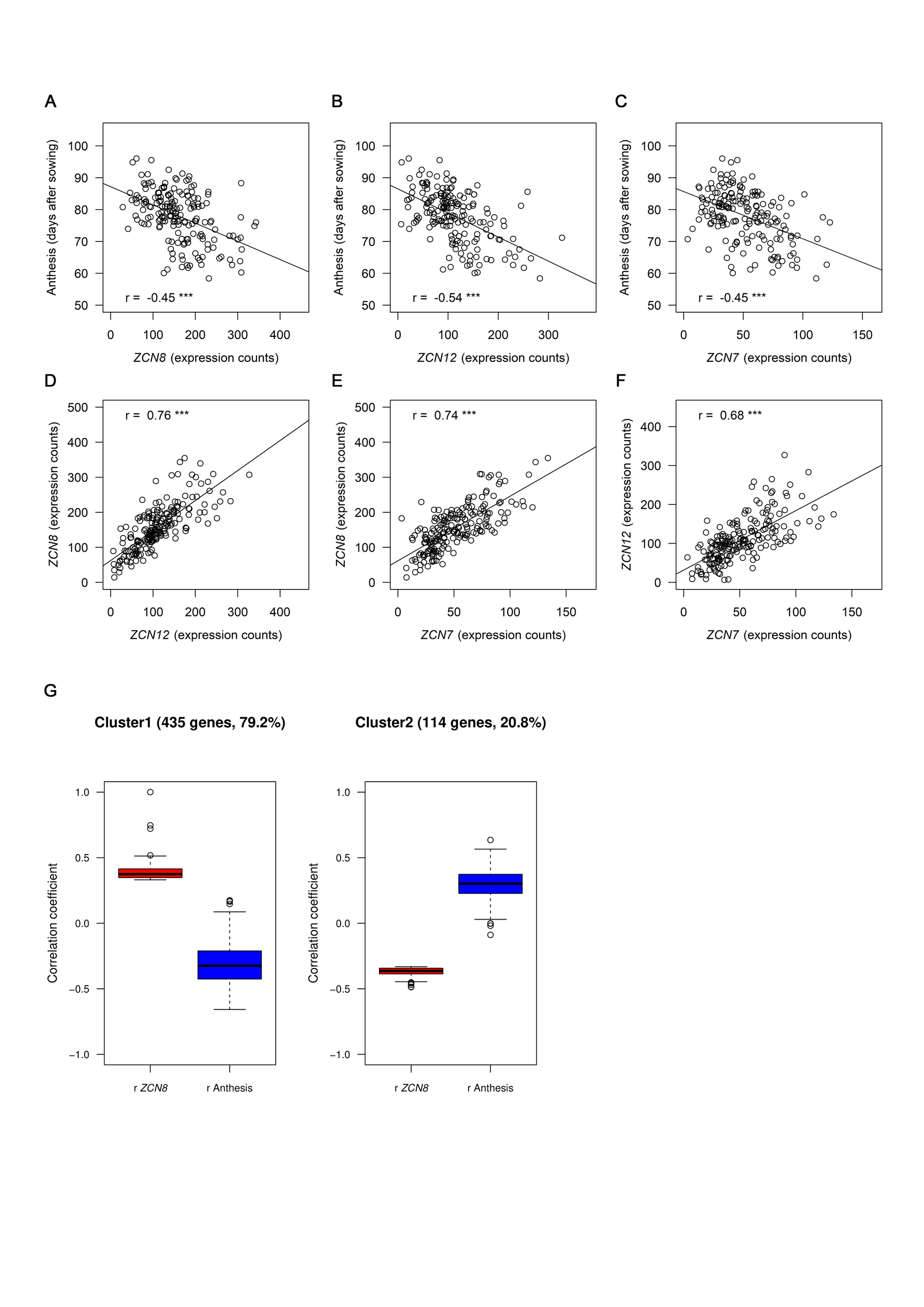

Supplement: S9 Fig — (A to C) Re-analysis of publicly available data profiling whole genome expression variations from different maize varieties. Comparisons between anthesis and accumulation of respectively ZCN8 (A, n = 182 lines), ZCN12 (B, n = 180 lines) (B), and ZCN7 (C, n = 178 lines) (C). (D-F) Pairwise comparisons between ZCNs transcripts accumulation, respectively ZCN8 vs ZCN12 (D, n = 200), ZCN8 vs ZCN7 (E, n = 198), and ZCN12 vs ZCN7 (F, n = 198). Genotypic values (BLUPs) are displayed for anthesis, whereas individual values (no biological replicates) were reported for ZCN8, ZCN12 and ZCN7 accumulation. Pearson’s correlation coefficients are indicated with their significance (***, p < 10−4). (G) k-means cluster analysis on the 549 ZCN8-coregulated genes based on their degree of correlation with both ZCN8 expression and flowering time. (TIF) [file pgen.1008882.s009.tif]

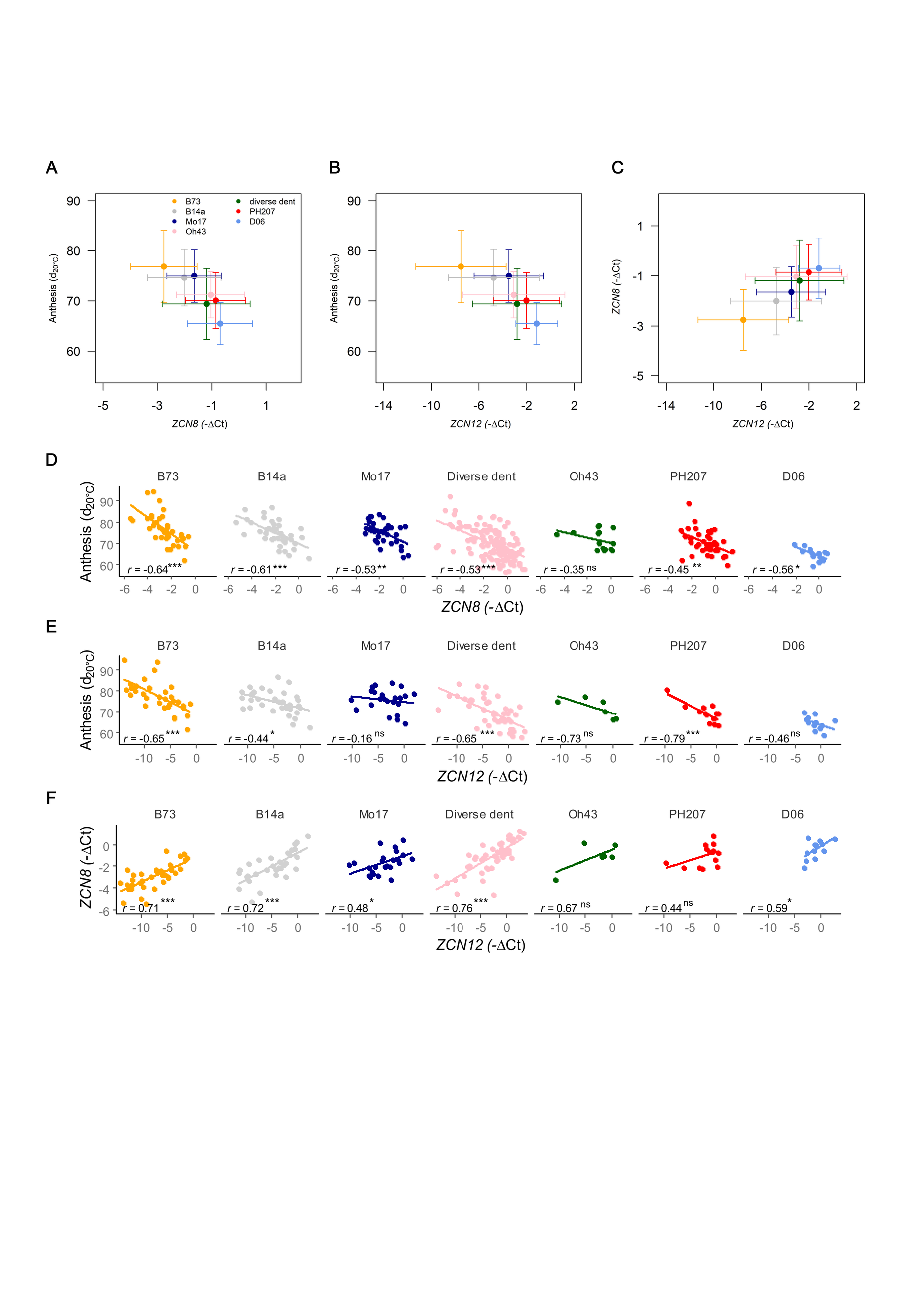

Supplement: S10 Fig — (A-C) Biplots of genetic group values for anthesis vs ZCN8 accumulation (A), anthesis vs ZCN12 accumulation (B), and ZCN8 vs ZCN12 accumulation (C). Means and standard deviations of genetic group values are calculated from the BLUEs in the platform experiment of 2014. Genetic groups are represented by the name of their founder and the colour code reflects flowering time phenology as in Fig 5A. D06: n = 14; PH207: n = 42; diverse dent: n = 128; Oh43: n = 16; Mo17: n = 35; B14a: n = 41; B73: n = 37. (D-F) Within-group relationship between anthesis and accumulation of respectively ZCN8 (D), ZCN12 (E) and accumulation of ZCN8 vs ZCN12 (F). (TIF) [file pgen.1008882.s010.tif]

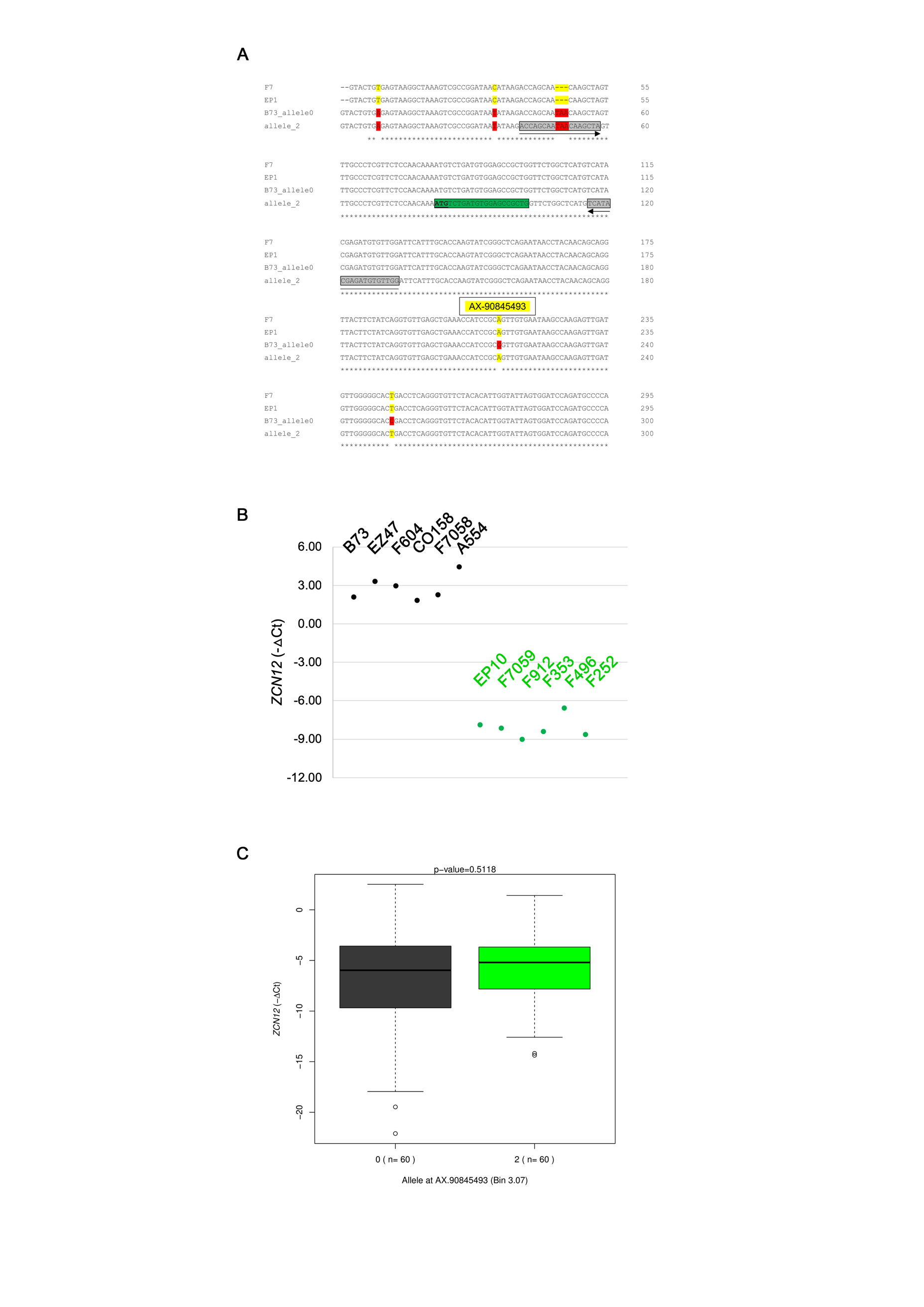

Supplement: S11 Fig — (A) Sequence alignment of the ZCN12 region targeted for amplification in different maize lines. Nucleotides highlighted in grey boxes correspond to forward and reverse primers, respectively whereas the green box corresponds to the probe. Nucleotides highlighted in yellow indicate polymorphisms. Marker AX-90845493 was used to differentiate the B73 from the alternative haplotype in our lines. (B) ZCN12 Taqman assay using genomic DNA derived from lines carrying the B73 allele (black) or the alternative allele (green). UCe amplification was used for normalization and to derive—ΔCt values. (C) ZCN8 transcript amplification performed on a reduced panel and with a different amplification assay. (TIF) [file pgen.1008882.s011.tif]

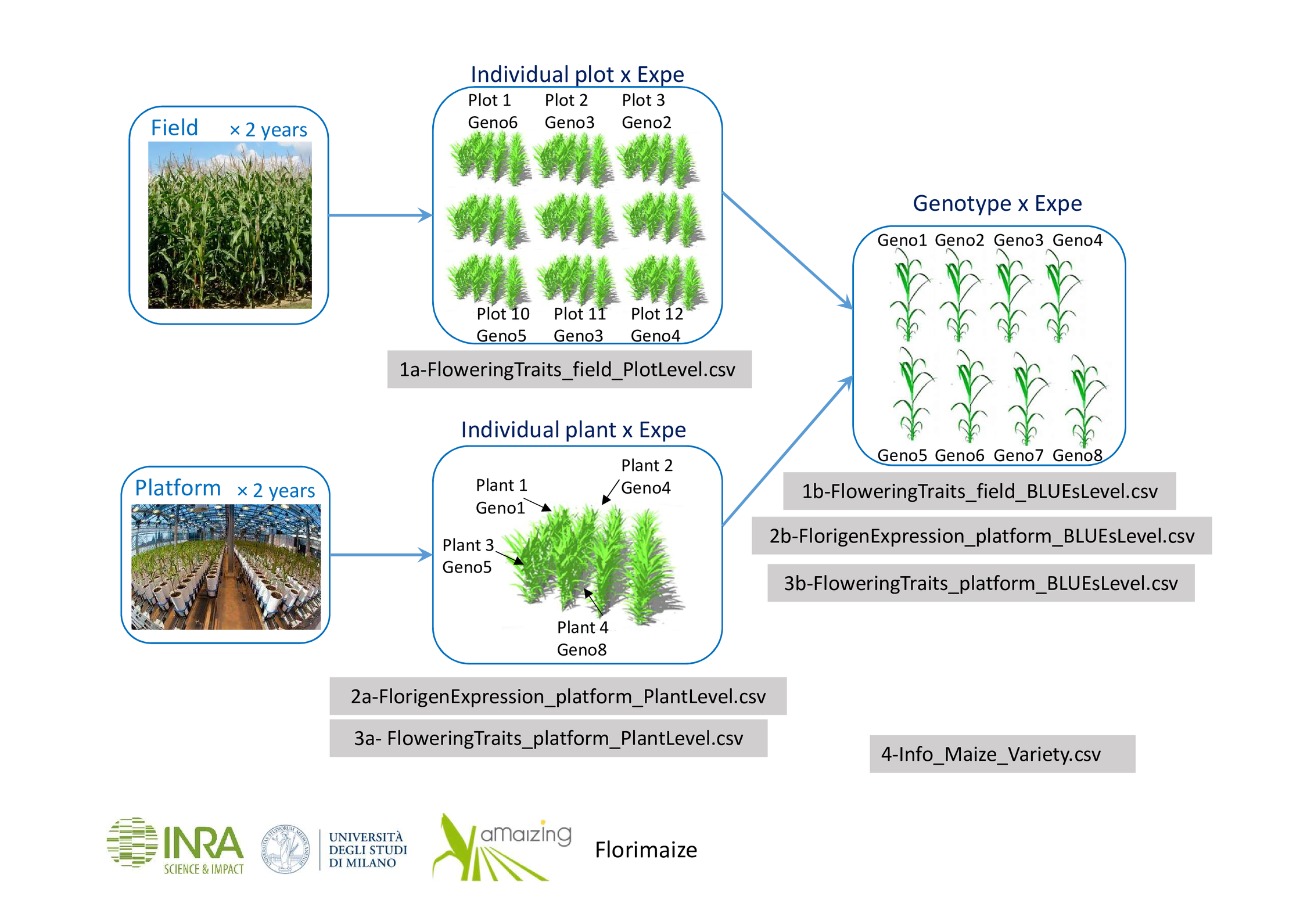

Supplement: S12 Fig — Phenotypic data at the plot level in each field experiment (1a) and at the genotypic level in each field experiment and for both field experiment together (1b). Transcripts data at the plant level in each platform experiment (2a) and at the genotypic level in each platform experiment (2b). Phenotypic data at the plant level in each platform experiment (3a) and at the genotypic level in each platform experiment (3b). Description of the genotypic material used (4). The maize 3D canopy is adapted from Pradal et al., [72]. The maize 3D plant is adapted from Fournier and Andrieu [73]. (TIF) [file pgen.1008882.s012.tif]
