# Supplementary material for: Maize adaptation across temperate climates was obtained via expression of two florigen genes
Source: PLoS Genet. 2020 Jul 16;16(7):e1008882. doi: 10.1371/journal.pgen.1008882 (PMC7386623; doi:10.1371/journal.pgen.1008882)
Supplement: Attachment — Submitted filename: PGENETICS-D-19-01500_reviewerF.pdf [file pgen.1008882.s021.pdf]

The authors present results showing that ZCN8 and ZCN12 are key regulators of flowering time in temperate maize. Although ZCN8 is a well-known regulator, the authors provide further evidence of its importance linking its expression levels in diverse materials with flowering time and leaf number. More importantly, the authors show strong evidence demonstrating that ZCN12 expression behaves similarly to ZCN8 and seem to share some common regulators (while ZCN7 does not). Overall, in my opinion this is a well written paper with enough evidence supporting their claims.

However, this reviewer has some concerns about data availability. Although stated otherwise in the submission, none of the original phenotypic or genotypic data is presented and important details about the germplasm evaluated (i.e. list of taxa) are missing. Consequently, my recommendation is acceptance with major revisions to include additional data and details.

1. In line 125 the authors mention an association panel of 327 lines with no further details. In the methods (l. 534) the initial panel from where samples were taken is cited, and later (l. 563) it is mentioned that only 327 were evaluated to fit the space requirements. But nowhere is explained how those were selected or what are the final taxa on the experiment. Similarly, the panel cited in line 170, line 211, or line 214. This could all mostly be solved by including the phenotypic files (raw and BLUEs) for the different traits/subpanels as supplemental (or with associated DOI)

2. Total Leaf number (l. 130) correlates with flowering time but it is not flowering time. And it is a trait that can be measured in different ways since maize plants can lose leaves during development. How was it measured? Visible leaves at the end of cycle or total leave number (including lost)?

3. In line 135, a broad sense heritability up to 0.95 is reported. But the corresponding supplemental table has values up to 0.97. In general, the values reported for broad sense heritability on table S1 seem quite high, even for a highly heritable trait like FT. But since the original data is not provided this result cannot be replicated/confirmed. In addition, why is not the combined trait across environments analyzed instead of the by environment data (or at least combine all field and all platform)? QTL results would be cleaner and easier to follow.

4. In the "Evidence for selection" section (l. 353), while the Fst results presented at the whole population levels seem ok, this reviewer has problems with them when dividing the samples into groups as the number of individuals in two of the early breeding groups (Oh43 and D06) seem low (<20-30 individuals). One of the biggest selection blocks (Chr. 8 ~134 Mb) was detected in the B73 vs. Oh43 group, however all three correlations for this population with ZCN levels (S11-A-C) are non-significant. In addition, although it is possible that there is a FT QTL for this subset of Oh43 related lines, it has been reported that B73xOh43 do not show a segregating FT effect in that region (Buckler et al. 2009). These results may suggest lack of power to differentiate selection from drift.

5. The authors use the published data from Kremling et al 2018 to confirm some of their findings (l. 681) a) Why was only F7 discarded and no other flints? b) why only look at mature leaves and not L3 base or tips as seems closer to the data presented in this paper? c) The path does not point to the dataset. According to a README in the folder, the dataset is now available at https://doi.org/10.25739/3ph0-x861 . Please modify the citation.

6. In the discussion (l. 432) the authors mentioned that they can not identify cis signals for ZCN regulation. a) How is cis defined (+/-5Kb?) b) Are the number of SNPs analyzed (~750K) enough for this

conclusion? Same as previously, the Kremling et al dataset could be used to confirm/deny this statement. Hapmap data could also be used to explore the haloptypes of the different groups within the vicinity of the ZCNs to support the lack of diversity statement (l. 434).

7. The authors inconsistently use Maize (for example l.405) and maize (for example l.400). Please change all to maize.

8. Line 249 "(see methods)" should not be used (as it is not used anywhere else).

9. Figure 5D would be easier to follow if colored by early-late allele, not 012 or B73.
