# Supplementary material for: Maize adaptation across temperate climates was obtained via expression of two florigen genes
Source: PLoS Genet. 2020 Jul 16;16(7):e1008882. doi: 10.1371/journal.pgen.1008882 (PMC7386623; doi:10.1371/journal.pgen.1008882)
Supplement: Attachment — Submitted filename: Response to reviewers.docx [file pgen.1008882.s022.docx]

*Below we address the criticisms raised by the three reviewers.*

**Reviewer #1**: The authors present results showing that ZCN8 and ZCN12 are key regulators of flowering time in temperate maize. Although ZCN8 is a well-known regulator, the authors provide further evidence of its importance linking its expression levels in diverse materials with flowering time and leaf number. More importantly, the authors show strong evidence demonstrating that ZCN12 expression behaves similarly to ZCN8 and seem to share some common regulators (while ZCN7 does not). Overall, in my opinion this is a well written paper with enough evidence supporting their claims.

However, this reviewer has some concerns about data availability. Although stated otherwise in the submission, none of the original phenotypic or genotypic data is presented and important details about the germplasm evaluated (i.e. list of taxa) are missing. Consequently, my recommendation is acceptance with major revisions to include additional data and details.

*Thank you for your positive evaluation and recommendations.*

*All data supporting our analyses have now been made publicly available (see data availability section). Two distinct datasets have been created at data.inra.fr (GnPis information system) and can be consulted as follows:*

*-Phenotypes (flowering related traits in the field and platform experiments, and florigen expression in the platform experiments) are accessible at* [*https://data.inra.fr/dataset.xhtml?persistentId=doi:10.15454/XTHN7I*](https://data.inra.fr/dataset.xhtml?persistentId=doi:10.15454/XTHN7I)*. This includes raw data and BLUEs, and a description of the germplasm (variety and accession identifiers, holding institution, genetic groups) is also provided.*

*-Genotypes (genotyping matrix and SNP description) are accessible at* [*https://data.inra.fr/dataset.xhtml?persistentId=doi:10.15454/GAHEU0*](https://data.inra.fr/dataset.xhtml?persistentId=doi:10.15454/GAHEU0)

*Furthermore, we felt it could be helpful to have a graphical overview of the experimental design of this study. This information has now been added as Supplementary material S12.*

1. In line 125 the authors mention an association panel of 327 lines with no further details. In the methods (l. 534) the initial panel from where samples were taken is cited, and later (l. 563) it is mentioned that only 327 were evaluated to fit the space requirements. But nowhere is explained how those were selected or what are the final taxa on the experiment. Similarly, the panel cited in line 170, line 211, or line 214. This could all mostly be solved by including the phenotypic files (**raw and BLUEs**) for the different traits/subpanels as supplemental (or with associated DOI)

*Agree. We have now uploaded all the information including which lines are present in each experiment at the link mentioned above. The initial panel of 354 individuals was designed for genome wide association studies and to apply genomic prediction for complex traits (in the framework of the DROPS and AMAIZING projects). It consists of historical and representative breeding dent lines from the main founder groups of modern maize. To fit space requirements, subsets had to be constituted for the platform experiments. Subpanels of respectively 327 and 276 were optimized according to Rincent et al. (2012) algorithm that allows to maximise genetic information by discarding individuals that might be more easily predictable. This is now described in the method section (Flowering-related traits measurements in the platform).*

2. Total Leaf number (l. 130) correlates with flowering time but it is not flowering time. And it is a trait that can be measured in different ways since maize plants can lose leaves during development. How was it measured? Visible leaves at the end of cycle or total leave number (including lost)?

*We scored total leaves (including lost) at the end of the cycle. We labelled leaves with a marker pen during plant development (e.g. L4, L7 etc.) so to account for early leaves that were lost during development. These details were added to the method section for clarity (line 325 and -339: Male (anthesis) and female (silking) flowering dates, as well as the total number of leaves (including the ones lost) produced were measured for all the lines.).*

3. In line 135, a broad sense heritability up to 0.95 is reported. But the corresponding supplemental table has values up to 0.97. In general, the values reported for broad sense heritability on table S1 seem quite high, even for a highly heritable trait like FT. But since the original data is not provided this result cannot be replicated/confirmed.

*We agree that our broad-sense heritability values (which were calculated as H²= VarG/(VarG VarSpatial + VarRes/n), with n=5 plants per line) for the flowering-related traits are high. This is consistent with the high genotypic variance indicated in the table. We have added the calculation in the legend of the table for clarity. We have also now added an interval for broad-sense H² (H²low and H²high) with calculations detailed in the legend of Table 1. The strong genotypic control on these traits is also confirmed by the high narrow-sense heritability also reported in the table, calculated from the BLUEs with the R-package Heritability (Kruijer et al., 2014). We have now replaced all mentions of heritability in the text by the narrow sense values whose calculation is less controversial.*

In addition, why is not the combined trait across environments analyzed instead of the by environment data (or at least combine all field and all platform)? QTL results would be cleaner and easier to follow.

*For the field experiments, we do present the results obtained from BLUEs calculated over the two years (e.g. in figs 1C, 4B and supplemental figures). However, for the platform data, a smaller subset was assessed on the second year (276 lines in 2015) as compared to the first year (327 in 2014). We thus consider the 2014 experiment on 327 lines as the main platform result and working on multi-year BLUEs would result in the loss of lines absent from the 2015 experiment (or loss of accuracy for these lines). We thus present the results obtained in each year respectively. We have tried to make the QTL results section smoother and have oriented the main focus on the platform experiment conducted in the first year.*

4. In the “Evidence for selection” section (l. 353), while the Fst results presented at the whole population levels seem ok, this reviewer has problems with them when dividing the samples into groups as the number of individuals in two of the early breeding groups (Oh43 and D06) seem low (<20-30 individuals). One of the biggest selection blocks (Chr. 8 ~134 Mb) was detected in the B73 vs. Oh43 group, however all three correlations for this population with ZCN levels (S11-A-C) are non-significant. In addition, although it is possible that there is a FT QTL for this subset of Oh43 related lines, it has been reported that B73xOh43 do not show a segregating FT effect in that region (Buckler et al. 2009). These results may suggest lack of power to differentiate selection from drift.

*We agree with the reviewer and for this reason we have decided to show our results on the whole population and leave out pairwise comparisons. This data is now shown in Figure 5B (and related Table S7).*

5. The authors use the published data from Kremling et al 2018 to confirm some of their findings (l. 681) a) Why was only F7 discarded and no other flints? b) why only look at mature leaves and not L3 base or tips as seems closer to the data presented in this paper? c) The path does not point to the dataset. According to a README in the folder, the dataset is now available at https://doi.org/10.25739/3ph0- x861 . Please modify the citation.

*a) We initially thought F7 was the only flint in this dataset but have realized thanks to the reviewer’s comment that this is not the case. Because the detailed information of origin is not provided in that dataset, we have now chosen to only filter extreme outliers for gene expression using the criteria detailed in the M&M: respectively ZCN8 > 400, ZCN12 > 350 and ZCN7 > 150 expression counts.*

*b) We did focus on the tissue “L3 tip” initially, but we found that our target genes were poorly expressed at that stage. For ZCN8, expression counts were available for about 50% of the lines described and an even more reduced representation was observed for ZCN12 and 7 expression. Moreover, expression counts for ZCN8 at L3 tip were often 100-fold reduced compared to those observed in mature leaves. This heterogeneity in expression values and low representation led us to opt for mature leaves (now detailed in the M&M line 467: We used data for post-sexual maturity leaves during the day (LMAD) because expression counts for ZCN7/8/12 at earlier stages (e.g. leaf 3) were extremely low and not available for all lines.).*

*c) Thanks, we have now corrected the reference to the path to this dataset.*

6. In the discussion (l. 432) the authors mentioned that they can not identify cis signals for ZCN regulation. a) How is cis defined (+/-5Kb?) b) Are the number of SNPs analyzed (~750K) enough for this conclusion? Same as previously, the Kremling et al dataset could be used to confirm/deny this statement. Hapmap data could also be used to explore the haloptypes of the different groups within the vicinity of the ZCNs to support the lack of diversity statement (l. 434).

*a) Our conclusion is based on lack of eQTLs associated with the promoter region containing regulatory ZCN8 elements (as defined by the Guo et al. 2018 study). The closest eQTL signals we detect is located 10Mbps downstream of ZCN8 – thus unlikely to act in cis).*

*b) Thank you for this insightful observation. A flowering time QTL was indeed detected in the putative 5’ regulatory region of ZCN8 (approx. 1.7 kb upstream of ZCN8). Moreover, while we could detect significant SNPs variation in a 5kb region upstream of ZCN8 no association with ZCN8 expression was found. It is unlikely that the absence of a cis eQTL for ZCN8 depends on low coverage of the genetic diversity because these SNPs have been discovered on a large set of diverse inbred lines (17,000 and 22 lines for 6 GBS and 3 Affymetrix Axiom markers, respectively). Thus, we discarded the “lack of diversity” statement in this revised version. Although we cannot exclude that the absence of a cis QTL for ZCN8 derived from transient regulations or allelic interactions, the most parsimonious conclusion is that ZCN8 is regulated by trans-acting factors in the studied germplasm. Following the reviewer advice, we now clarify this point in our discussion see Lines 236-251.*

7. The authors inconsistently use Maize (for example l.405) and maize (for example l.400). Please change all to maize.

*Thank you for pointing out this inconsistency, we have now corrected this.*

8. Line 249 “(see methods)” should not be used (as it is not used anywhere else).

*We have now removed this inconsistency*.

9. Figure 5D would be easier to follow if colored by early-late allele, not 012 or B73.

*We have changed the general layout of Figure 5. The current figure 5A shows the SNPs distribution across the different maize groups. To improve readability, we color-coded the allele effect (late vs early), we ranked maize groups in a top to bottom fashion to reflect the flowering time phenology, whereas the color code of each group corresponds to the one used in Figure S10. This information is now reported in the legend. We hope the graphical representation is now clearer.*

**Reviewer #2**: The paper by Castelletti et al combines diversity panel analysis using a wide range of maize inbreds and large scale expression analysis to further our understanding of the genes that control the floral transition. In particular they examine expression in leaf tissue and therefore are focused on long distance signalling molecules, or florigens. Only the ZCN8 gene has been identified as a veritable maize florigen, however many plant species reportedly have multiple florigen genes. The focus here is on ZCN8 and a putative florigen, ZCN12. The researchers use expression profiling and transgenic analysis to show that ZCN12 encodes a florigen and, more relevant to this study, ZCN8 and 12 cooperate to control flowering in temperate maize, which is essentially day-neutral and therefore not dependent on photoperiod-induced flowering (i.e. autonomous). Further, they suggest that ZCN7, a putative syntenic paralog of ZCN8, may act upstream of both ZCN8 and 12 to cause flowering. A better understanding of the elements underlying flowering in autonomous maize is welcome, as this is a particularly intractable problem and not s easily addressed as has been done in photoperiod dependent plants.

Overall the paper is well written and the ideas behind the analysis are laid out fairly clearly (with the exceptions noted below). There is perhaps a sight over-interpretation of some of the data, in particular with respect to the model building based on expression analysis. In fact the overarching conclusions regarding how these genes may cooperate to control flowering is highly dependent on speculative modeling, and therefore the underlying conclusions are not as robust as they could be. Even so, given the complexity of the maize genome and the rapid evolution of maize from photoperiod dependent to autonomous flowering, the results presented here at least provide some inroads for further testing to the proposed models.

Some specific points:

1 Expression changes in the ZCN8 gene in autonomous maize are reported to be fairly constant, although it accumulates as the plants age. The authors take great care in explaining the experimental design, which is appreciated because too many studies do not take this into account.

*Thank you for your positive feedback.*

2. The diversity panel examined here no doubt contains maize from a fairly large geographic range. Previous studies (e.g. Cole et al 2010) show that different maize lines exhibit a range of photoperiod responses (i.e., only a few northern lines are truly day-neutral). Therefore, So the question is, does this have an impact on the interpretation of the data? That is, it seems the more photoperiod-sensitive maize is, the greater the expression changes in florigen genes (as was shown for ZCN8). If so, do the authors need to take this into consideration when proposing models? The current model assumes “desensitization to photoperiodic cures” (lines 38-39), but they should acknowledge that maize flowering is not “either one or the other”.

*We did not assess photoperiod sensitivity for individual lines represented in our panel. We note that according to Cole et al while “B73 and B97 had significant but smaller photoperiod responses for height and leaf number” they “showed no significant photoperiod responses for flowering time”. Since these lines are among the latest in maturity in our panel, we assume that photoperiodic signals play a rather small effect in our experiment.*

*While we agree there is still the possibility that vestiges of a photoperiodic response may contribute to repressing florigen expression under long day conditions (as used in our panel), we do not find strong evidence to support this idea. We specifically comment on this in our revised discussion: (lines 249: We did not detect any obvious candidate genes associated with photoperiodic signalling (e.g. ZmCCT10), suggesting its small contribution in ZCN8/12 regulation in our panel.).*

3. This study focuses on 3 florigen genes 7,8 and 12), one of which is verified here and elsewhere. But are these the only one and should they take into consideration other possible florigens? They might want to mention the findings of Stephenson et al (2019) where they report that ZCN14, 15 and 18 have florigen activity as well. Of course this would increase the complexity of the model, but is there a reason to not include them?

*The point is well noted. Due to available resources we decided to focus on the three florigen genes i) whose role was verified via transgenic approaches in maize (e.g. ZCN8) ii) that were activated upon photoperiod shifts in tropical maize as in Meng et al. (e.g. ZCN8/ZCN12) iii) were the product of genome duplication and hence possibly redundant (ZCN7/8). We did consider extra experiments on other florigen genes that fulfilled the above criteria but re-analysis of the Kremling et al. dataset revealed very little correlation between florigens like ZCN14,15,18 and flowering. This does not rule out their contribution in the flowering process per se. Rather, it indicates little variability that can be associated with flowering in natural maize.*

4. They says ZCN8 expression is negatively correlated with flowering time. Although technically correct, this is confusing and not normally used. True, as ZCN8 expression increases the days to flowering decreases, but nonetheless it would be easier to understand if they state clearly that ZCN8 expression has a positive effect on flowering.

*Agree and we rephrased it to clarify the concept. (Line 133: Anthesis time was significantly correlated with the BLUEs of ZCN8 (r = -0.6, Fig 1B) and so was the total leaf number (r = -0.65, Supplementary Fig. 5A)).*

5. Lines 107-108: The authors state that ZCN8 decrease causes a strong delay in flowering, as reported by others. But this is an overstatement. The only report, as far as I know, of ZCN8 knockdown was by Meng et al (2011), and they found only slight delay in flowering (3 or 4 leaves). Whereas mutants such as dlf1 and id1 produce many more leaves and show a true strong effect. Also of the cited papers indicating a strong effect on flowering, only Meng et al, 2019 show these data; the other papers, (Stephenson et al 2019 and Liang et al, 2019) do not as far as I can tell.

*That is correct. We rephrased to reflect more accurately the contribution of ZCN8 in flowering compared with id1 or dlf1 (line 91: Two elements challenge this view: (i) the downregulation of ZCN8 in temperate maize leads to a significant delay in flowering time (albeit less prominent compared to id1 or dlf1 mutants)).*

*Reference to the Liang et al paper is because they reported loss-of-function for ZCN8 generated via Crispr-Cas9. Reference to the Stephenson paper is because overexpression of ZmCCT10 causes a strong delay in the early maturity maize Gaspe’ Flint which is associated with repression in ZCN8 levels. Although indirectly, it points to a prominent role of ZCN8 in maize flowering.*

6. The co-regulation of ZCN8 and 12 and how they might interact is one of the novel findings of this paper. This is partly based on the SEM analysis presented in Figure 3. I have to confess that I do not understand how this model was built. Although explained in the Methods section, I had a hard time understanding how the pathways were tested and why A and B were rejected. Perhaps those well-versed in this type of modeling have a better understanding of this type of analysis, but I think others would benefit from a more lucid explanation of how this was put together and what it means “in the real world” of floral induction.

*We acknowledge that readers might not all be familiar with SEM and we have thus added more elements to the Material and Methods section. In-depth description of SEM can be found in the three references cited in the M&M (Shipley, 2016; Pugesek et al., 2004; Lamb et al., 2011).*

*We used SEM because, rather than an analysis of the effects on a single variable of one or more independent variables, it allows a multivariate analysis of the relationships between many variables with specification of a network.*

*The inputs to a SEM are (1) a variance-covariance matrix between the variables in a dataset, and (2) an initial path diagram hypothesizing the causal relationships among the variables.*

*Practically this means that to run the analysis, we (1) used the dataset with BLUEs of the 4 traits considered (anthesis, ZCN8, ZCN12 and ZCN7 expression) from which the variance-covariance matrix was deduced; and (2) wrote different models corresponding to different network structures to be tested. One model is a set of equations that define the relationships between the variables. We based the network structures to be tested on (i) prior knowledge from the literature: anthesis was placed downstream of genes expression (flowering is a consequence of florigens expression), (ii) then testing for all possible network structures between the 3 florigens acting upstream of anthesis.*

*The structural equation model fit is then evaluated by comparing the model-implied variance-covariance matrix to the observed variance-covariance matrix. The initial path diagram is translated into a set of linear equations describing the relationships (slopes, intercepts, variances, etc.) that imply an expected pattern to the variance-covariance matrix. Parameter values for the linear equations that minimize the deviation between the observed and model-implied covariances are estimated using maximum likelihood or other methods. Tests are then applied to determine whether the covariance structures implied by the model adequately fit the actual covariance structures of the data based on a discrepancy function. The most common test of model fit is a chi-squared test with a null hypothesis of adequate fit and an alternative hypothesis of inadequate fit. A non-significant (P>0.05) test result indicates adequate model fit, or more generally that the data are consistent with the initial causal hypothesis (path diagram), indicating that no important paths have been omitted from the model.*

*This method has been successfully used in other physiological studies and, although purely based on statistics, it helped deciphering important processes such as the control of leaf development (Tisné et al., 2008 Plant Physiology, DOI:* [*https://doi.org/10.1104/pp.108.124271*](https://doi.org/10.1104/pp.108.124271)*) and yield elaboration (Lamb et al., 2011, Can J Plant Sci., DOI:10.4141/cjps2010-035).*

*We have tested multiple path diagrams accounting for different sequence of actions of our florigens (as reported in the principal and supplemental figure) and all those where ZCN12 had an indirect effect on flowering were rejected. In other terms, ‘cascade’ models where ZCN12 expression determines ZCN8 expression which in turn determines flowering, were rejected. We thus believe that this approach sheds additional lights suggesting that while ZCN7 may act upstream of* *ZCN8 and ZCN12 or through other pathways (e.g. ZCN7 expression may cascade into the transcriptional activation of ZCN8 and ZCN12 or act independently), ZCN8 and ZCN12 regulate flowering directly (e.g upon their transcriptional activation they act redundantly to activate flowering (presumably by moving at the shoot apex).*

(by the way, line 246: “synergic” should be “synergistic”)

*Thank you for pointing out this error. This sentence was eliminated.*

7. Given the predicted redundant roles played by ZCN8 and 12, it would be interesting to see whether a double knock out is very late flowering. Not a trivial experiment in maize, but worth thinking about.

*Thank you for this suggestion. It is clearly an important angle for future research.*

**Reviewer #3**: The manuscript by Castelletti et al deals with florigene (ZCN8) and florigene-like/related gene expression across a large maize panel. The objective was to investigate how such florigenes are expressed and regulated in temperate (= mostly photoperiod insensitive) maize materials. Wild maize (= teosinte), and most maize landraces are photoperiod sensitive, thus they are strongly flowering-delayed when grown in temperate latitudes, while modern temperate maize cultivars or hybrids appear mostly photoperiod insensitive. The expansion of maize from tropical latitudes to northern or southern environments must have required dramatic changes in photoperiod sensitivity and flowering time, however how these changes involved florigene(s) expression changes is indeed not understood yet. The topic is of substantial scientific and practical (breeding) interest. The experimental strategy deployed by the authors was in my opinion essentially correct and potentially informative. The technical tools applied were appropriate. It is worth mentioning that phenotypic data and leaf samples for gene expression analysis were collected in a large, well replicated experiment which was carried out in a state-of-the-art phenomics facility.

Main results

A. identification of a large range of variation of ZCN8 gene expression and strong correlation between ZN8 and flowering time in temperate maize materials, which suggested that flowering time variation involves variation of ZCN expression.

B. Similar results for ZCN12, which was also shown to act as flowering inducer in Arabidopsis when overexpressed.

C. A strong coincidence between ZCN8 and ZCN12 eQTLs and flowering time QTL.
D. Lack of detection of trends of co-expression between ZCNs and genes corresponding to eQTL for ZCNs (Results lines 327-329; Discussion lines 448-451).

Comments.
1. A major problem for this manuscript is the large number of experiments and thus the consequent necessity for the authors to present and discuss many results. I have to admit that, for the RESULTS section, it was not always easy to follow the experiments succession and the underlying rationale. Therefore I would strongly suggest the authors to subdivide RESULTS paragraphs in shorter ones, corresponding to single experiments and/or single hypothesis under testing. In my opinion, text length can be safely reduced. A CONCLUSIONS section would probably help too.

*We have taken on board these valuable suggestions and revised the manuscript accordingly. The text has been shortened and extensively rewritten in order to convey more clearly both the results and the underlying interpretation. We have now organised the results in five subsections (as opposed to four ones in the previous version) according to the specific hypothesis that is discussed.*

*Previous subsection titles:*

*i) ZCN8 levels are highly correlated to flowering time in temperate maize*

*ii) ZCN12 is a positive regulator of maize flowering and it is co-regulated with ZCN8*

*iii) ZCN8 and ZCN12 regulators map to region controlling maize flowering time*

*iv) Evidence for selection at ZCN8 and ZCN12 eQTLs to control maize flowering time*

*Current subsection titles:*

*i) ZCN8 levels highly correlate with flowering time in temperate maize*

*ii) ZCN12 is a novel regulator of maize flowering and it is co-expressed with ZCN8*

*iii) Direct roles of ZCN12 and ZCN8 in the control of flowering time*

*iv) ZCN8 and ZCN12 regulators map to region controlling maize flowering time*

*v) Signs of selection at ZCN8 and ZCN12 eQTLs to control maize flowering time*

*A Conclusions section has been included in the revised version.*

*We have also now added a supplemental figure (S12 Fig) which provides an outline of the experiments and datasets used.*

2. Some RESULTS parts could go either in MAT and MET of in DISCUSSION. For instance, lines c. 128-132 and 160-166 reported technical details typical of MAT and MET. Lines 193-195 can probably be deleted. Line 198-199 seem to be a contradiction of the overall message of this message, so please check and perhaps delete.

*Thank you for these valuable suggestions which were included in the revised version.*

*The sentences in lines 128-132 and 160-166 were discarded because deemed unnecessary in the Results section. Lines 193-195 were also dismissed.*

*We agree that the sentence in lines 198-199 was not consistent with our findings and we therefore removed it.*

3. Concerning point D above, I wonder whether strong post-transcriptional regulation (eg. by miRNA) or post translational regulation (at protein level) could be listed as possible causes behind lack of correlation. At least for ZmRap2.7, it was clearly shown that besides being regulated by a long distance regulatory element, it was also post-transcriptionally regulated by miR172.

*We agree and we have now acknowledged this possibility in the discussion (lines 255: For example, microRNA172s are known to interfere with the translation of APETALA2 genes [37] (such as Rap2.7), indicating that transcript accumulation of Rap2.7 may not reflect its gene product levels. ).*

4. Fig. 4 and related text. I suggest to compute and show LD relationships for the whole region (ie. from left hand side to right end side), and not just for three somewhat arbitrarily chosen regions. Long range LD relationships would probably prevent drawing any conclusions about the possibility to locate a single causative variants, or any other general trends. Additionally, if appropriate, the authors should recognize that long LD at this locus was already observed previously using a different maize panel (Hirsch et al. 2014, cited in this manuscript as [24]).

*Thank you for these valuable remarks. We now represent the LD along the region in the new figures 4 and S8. Indeed, while we detect LD between the best hits, we do not observe significant long-distance LD; the three highlighted regions had low LD between them on average.*

*We now properly acknowledge previous studies on the role of the region highlighted in Fig 4 (and candidate loci therein contained):*

*Line 79: Both ZmMADS69 and Rap2.7 map within well-characterized QTLs for flowering time suggesting that allelic variations at these genes control part of the diversity of flowering time of temperate maize (Salvi et al., 2007; Castelletti et al., 2014; Hirsch et al., 2014).*

*Line 188: In the Bin 3.05 hotspot region known for flowering time regulation (Hirsch et al., 2014; Negro et al., 2019).*

5. Maize should be maize (no capital letter).

*Thank you for pointing out this inconsistency. We have now corrected this.*

6. QTL or QTLs (in case of multiple loci)? There is no consistency across manuscript.

*Thank you for pointing out this inconsistency. We have now corrected this.*

7. I have the impression the title might contain mistakes. Please consider that ‘explain’ could be ‘explains’ and ‘variations’ could be ‘variation’.

*Thank you for pointing out this mistake. The title has been modified in the revised version (Current title: Maize adaptation across temperate climates was obtained via expression of two florigen genes).*
